# The Clinical Significance and Potential Role of Cathepsin S in IgA Nephropathy

**DOI:** 10.3389/fped.2021.631473

**Published:** 2021-04-12

**Authors:** Jingying Zhao, Yongchang Yang, Yubin Wu

**Affiliations:** Department of Pediatrics, Shengjing Hospital of China Medical University, Shenyang, China

**Keywords:** IgA nephropathy, CTSS, Mesangial Cell Proliferation, CTSS inhibitor 2, Pediatrics

## Abstract

**Objective:** Cathepsin S (CTSS) is an important lysosomal cysteine protease. This study aimed at investigating the clinical significance of CTSS and underlying mechanism in immunoglobulin A nephropathy (IgAN).

**Methods:** This study recruited 25 children with IgAN and age-matched controls and their serum CTSS levels were measured by enzyme-linked immunosorbent assay (ELISA). Following induction of IgAN in rats, their kidney CTSS expression, IgA accumulation and serum CTSS were characterized by immunohistochemistry, immunofluorescence, and ELISA. The impact of IgA1 aggregates on the proliferation of human mesangial cells (HMCs) was determined by Cell Counting Kit-8 and Western blot analysis of Ki67.

**Results:** Compared to the non-IgAN controls, significantly up-regulated CTSS expression was detected in the renal tissues, particularly in the glomerular mesangium and tubular epithelial cells of IgAN patients, accompanied by higher levels of serum CTSS (*P* < 0.05), which were correlated with the levels of 24-h-urine proteins and microalbumin and urine erythrocytes and grades of IgAN Lee's classification in children with IgAN (*P* < 0.01 for all). Following induction of IgAN, we detected inducible IgA accumulation and increased levels of CTSS expression in the glomerular mesangium and glomerular damages in rats, which were mitigated by LY3000328, a CTSS-specific inhibitor. Treatment with LY3000328 significantly mitigated the Ki67 expression in the kidney of IgAN rats (*P* < 0.01) and significantly minimized the IgA1 aggregate-stimulated proliferation of HMCs and their Ki67 expression *in vitro* (*P* < 0.01).

**Conclusions:** CTSS promoted the proliferation of glomerular mesangial cells, contributing to the pathogenesis of IgAN and may be a new therapeutic target for intervention of aberrant mesangial cell proliferation during the process of IgAN.

## Introduction

Immunoglobulin A nephropathy (IgAN), also known as Berger's disease, is one of the most common glomerulonephritis worldwide. Approximatively, 15–25% of IgAN patients progress to end stage renal disease (ESRD) within 10 years after diagnosis (1). Pathologically, IgAN is characterized by predominant IgA and extracellular matrix deposition in the glomerular mesangium, accompanied by mesangial cell proliferation with matrix expansion, focal necrosis, segmental sclerosis, and crescent formation in the glomeruli (2). Clinically, IgAN patients display hematuresis with different levels of proteinuria, hypertension and kidney function impairments, and some IgAN patients develop glomerular sclerosis, even irreversible progression to ESRD. Hence, understanding the pathogenesis of IgAN and prevention of its progression into ESRD will be of high significance in management of IgAN patients.

Mesangial cells can regulate glomerular hemodynamics and are critical for renal glomerular function. However, little is known on the molecular pathways that regulate glomerular mesangial cell proliferation and matrix accumulation as well as inflammatory cell infiltration during the process of IgAN (3). Given that aberrant glomerular mesangial cell proliferation is crucial for the development of IgAN inhibition of glomerular mesangial cell proliferation may be an excellent strategy for delaying the pathogenic progression of IgAN.

Cysteine cathepsins are endolysosomal proteases (4). Cathepsin S (CTSS) is an important member of the lysosomal cysteine protease family and is important for antigen presentation, cytokine secretion, angiogenesis, and others (5). CTSS is highly expressed in the kidney tissues of patients with chronic kidney disease (CKD), lupus, diabetic nephropathy and other kidney diseases. Previous studies have shown that CTSS can promote tumor cell proliferation by activating the phosphoinositide 3-kinase (PI3K)/Akt and mitogen-activated protein kinase (MAPK) pathways (6, 7). CTSS can also stimulate the proliferation of human periodontal ligament cells (8). Currently, there is little information on how CTSS is expressed in patients with IgAN and on the role of CTSS in the pathogenesis of IgAN.

This study tested the hypothesis that CTSS might participate in the pathogenesis of IgAN by promoting glomerular mesangial cell proliferation and inhibition of CTSS might ameliorate the pathogenic process. We first characterized the expression of CTSS in children with IgAN and analyzed its association with clinical measures. Furthermore, we employed a rat model of IgAN to test the potential therapeutic effect of a specific CTSS inhibitor and potential mechanisms. Finally, we tested whether inhibition of CTSS could attenuate the IgA1-stimulated human mesangial cell (HMC) proliferation *in vitro* and potential mechanisms.

## Materials and Methods

### Patients

This study recruited 25 children with IgAN in the Department of Pediatric Nephrology, Shengjing Hospital of China Medical University from February 2017 to May 2019. Children with IgAN were diagnosed, based on clinical symptoms, laboratory examination, histological, and immunohistochemistric examination of renal biopsied tissues. Patients were diagnosed with IgAN if glomerular mesangial cell proliferation was present and IgA as the sole or predominant immunoglobulin deposition. The pathological grades of IgAN were evaluated by the Lee's classification. There were 3 in grade I, 16 in grade II, 4 in grade III, and 2 in grade IV. There were 13 cases with non-nephrotic syndrome level proteinuria (52%), and 12 cases with isolated hematuria type (48%, Table 1; Supplementary Table 1). Patients were excluded if she/he had another systemic disease, such as Schonlein–Henoch purpura, systemic lupus erythematosus, rheumatoid arthritis, diabetes mellitus, or received corticosteroids or other immunosuppressants before diagnosis. The control group included eight children, who underwent unilateral partial nephrectomy due to trauma or tumor in the same period and their surgical kidney tissue specimens were used for immunohistochemistry analysis. The serological control samples were obtained from 25 children, who underwent physical examination in our hospital (Table 1; Supplementary Tables 1, 2).

**Table 1:** The demographic and clinical characteristics of subjects.

	**IgAN patients (*n* = 25)**	**Controls (*n* = 25)**	**Controls (*n* = 8)**	***p-*value**
Gender, M/F ratio	1.5	1.08	1.7	ns
Age, years	10.6 ± 2.1	10.74 ± 2.5	10.25 ± 1.0	ns
Blood pressure systolic (mmHg)	103.7 ± 27.8		99.6 ± 16.2	ns
Blood pressure diastolic (mmHg)	69.1 ± 8.5		64.9 ± 7.9	ns
Isolated hematuria	12 (48%)			
Non-nephrotic syndrome level proteinuria	13 (52%)			
serum Scr (μmol/L)	59.12 ± 27.5		48.9 ± 3.26	ns
serum BUN (mmol/L)	5.56 ± 1.99		4.49 ± 1.18	ns
24-h-urine proteins(g)	0.65 ± 0.71			
Urinary microalbumin (mg/dL)	118 ± 131.2			
Urine erythrocytes/HP	288.3 ± 347.3			
Lee's classification (I + II)	19 (76%)			
Lee's classification(III + IV)	6 (24%)			

*Those 25 healthy control children were recruited from the Center of Physical Examination of our hospital. Those eight control non-IgAN patients underwent unilateral partial nephrectomy due to trauma or tumor*.

This study was approved by the Ethics Committee of Shengjing Hospital, China Medical University (2019PS441K). Written informed consent was signed by participants' parents.

### Experimental Animals and Model

Male Sprague Dawley (SD) rats weighing 180–220 g were obtained from Liaoning Changsheng Biotechnology (Benxi, China). The rats were housed in a specific pathogen-free room in the Experimental Center of Shengjing Hospital and allowed free access to food and water.

SD rats were randomly divided into three groups, the Control, IgAN, and CTSS inhibitor groups with 10 rats in each group. A rat model of IgAN was induced as described previously (9, 10). Briefly, SD rats were randomized and administrated with bovine serum albumin (BSA, 100 mg/ml in distilled water, 4 ml/kg, B2064, Sigma, St. Louis, USA) every other days by gavage for eight consecutive weeks and injected subcutaneously with 0.1 ml of CCl4 dissolved in 0.5 ml castor oil weekly for nine consecutive weeks as well as injected intravenously with 50 μg Lipopolysaccharide (LPS, L2880, Sigma) in saline at the 6th and 9th week post induction. Beginning on the 6th week post induction, the BSA-treated rats were randomized and injected intraperitoneally with 200 μl of saline (IgAN) or 200 μl saline containing 100 μg (0.5 μg/μl) LY3000328 (a specific inhibitor of CTSS, A3284, Apexbio, Houston, USA) daily for 3 weeks. The control rats received vehicles used in the experimental group. At 10 weeks post induction, peripheral blood samples were obtained from individual rats to prepare serum samples and stored at −80°C. All animals were euthanized. Their kidney tissues were dissected and fixed in 10% formalin overnight, followed by paraffin-embedded. The kidney tissues (4 μm) were subjected to Periodic acid–Schiff (PAS) staining.

This study was approved by the Ethics Committee of Shengjing Hospital, China Medical University (2019PS458K).

### Cell Culture and Treatment

HMCs were purchased from the China Center for Type Culture Collection, Shanghai and characterized by STR. HMCs were cultured in high glucose MEM medium containing 10% of fetal bovine serum (FBS, Hyclone, USA). Monomeric human IgA1 (Sigma) at 25 μg/ml was heated and aggregated at 65°C for 150 min on a dry plate-heater to obtain the aggregated IgA1 as previously described (11). The cells were pre-treated with vehicle or 10 μg/mL LY3000328 overnight and treated with 25 μg/mL IgA1 aggregates for 24 h (12).

### Immunohistochemical Staining

The levels of CTSS expression in the kidneys of individual rats were characterized by immunohistochemistry (13). Briefly, the kidney tissue sections (4 μm) were dewaxed, rehydrated, and subjected to the antigen retrieval process. The sections were incubated with rabbit anti-rat CTSS antibody (1:1,000, Novus Biologicals, USA) at 4°C overnight. After being washed, the bound antibodies were reacted with horseradish peroxidase (HRP)-labeled goat anti-rabbit IgG and visualized with DAB (3,3′-Diaminobenzidine), followed by counterstained with hematoxylin. The sections were photoimaged under a light microscope.

### Enzyme-Linked Immunosorbent Assay (ELISA)

The levels of serum CTSS in individual rats and human patients were quantified by ELISA using a commercially available ELISA kit (Abcam, USA), according to the manufacturer's instruction. The minimum detectable level was 4 pg/ml. Samples were tested in triplicate.

### Immunofluorescence

The levels of CTSS expression and IgA accumulation in the kidney tissues were characterized by immunofluorescence. Briefly, the kidney tissue sections (4 μm) were subjected to antigen retrieval process and probed with FITC-anti-IgAα (1:100, Abcam) or rabbit anti-CTSS (1:50, Santa Cruz, Biotech) overnight at 4°C. After being washed, the bound rabbit anti-CTSS antibodies were detected with Cy3-conjugated goat anti-rabbit IgG (1:200, Beyotime, China), followed by nuclearly stained with 4′,6-diamidino-2-phenylindole (DAPI, C1002, Beyotime). The immunofluorescent signals were observed under a fluorescent microscope (Nikon).

### Western Blotting

The relative levels of Ki67 expression in individual samples were measured by Western blotting. Briefly, the kidney tissues were homogenized and after centrifugation, the protein concentrations in the tissue lysates were determined using a commercial bicinchoninic acid (BCA) kit (ThermoScientific). Subsequently, the tissue lysates (20 μg/lane) were separated by sodium dodecyl sulfate–polyacrylamide gel electrophoresis (SDS-PAGE) on 12% gels and transferred to nitrocellulose membranes. The membranes were blocked with 5% fat-free dry milk in Tris-buffered saline, 0.1% Tween 20 (TBST) and probed with primary antibodies against Ki67 (1:100, ZSGH-Bio, Beijing, China) or β-actin overnight. The bound antibodies were detected with HRP-coupled goat anti-rabbit IgG (1:3,000 dilution, SE134, Solarbio, China), and visualizing with the enhanced chemiluminescent system (PE0010, Solarbio). The relative levels of Ki67 expression were quantified by densitometric scanning using ImageJ software.

### Quantitative Real-Time Polymerase Chain Reaction (qRT-PCR)

Total RNA was isolated from renal tissues and cells using total RNA isolating kit (Tiangen, Beijing, China) according to the manufacturer's introductions. The RNA samples were reversely transcribed into cDNA using an RT Primer (Genscript, Nanjing, China) and RNase inhibitor (DP418, Tiangen). RT-PCR reactions were carried out with the SYBR green PCR kit (SY1020, Solarbio) and specific primers of forward 5′CTGACCCTGATGAGAGTGAGGGA3′ and reverse 5′ACTCTGTAGGGTCGAGCAGG3′ for Ki67; forward 5′GACCTGACCTGCCGTCTAG3′ and reverse 5′AGGAGTGGGTGTCGCTGT3′ for glyceraldehyde-3-phosphate dehydrogenase (GAPDH). The relative levels of KI67 to the control GAPDH mRNA transcripts were analyzed by the 2^−ΔΔCt^ method.

### Cell Proliferation

Cell proliferation was detected by cell counting kit-8 (CCK-8) (Apexbio). Briefly, HMCs (2,000 cells /well) were cultured in 96-well-plates in triplicate for 0, 6, 12, 24, 36, and 48 h. Individual wells of cells were added with 10 μL of CCK-8 solution and cultured at 37°C for 2 h. The absorbance of individual wells was read at 450 nm in a microplate reader.

### Statistical Analysis

The statistical analysis was performed using SPSS 21.0. All data were analyzed in a blinded manner and are expressed as mean ± standard deviation (SD). Pairwise comparisons were performed by the Student *t*-test and the difference among multiple groups was determined by one-way analysis of variance and *post-hoc* the least significant difference test. The correlation was statistically analyzed by Pearson correlation tests. A *P*-value of < 0.05 was considered statistically significant.

## Results

### Increased Levels of CTSS Expression Are Detected Children With IgAN

**Figure 1:**
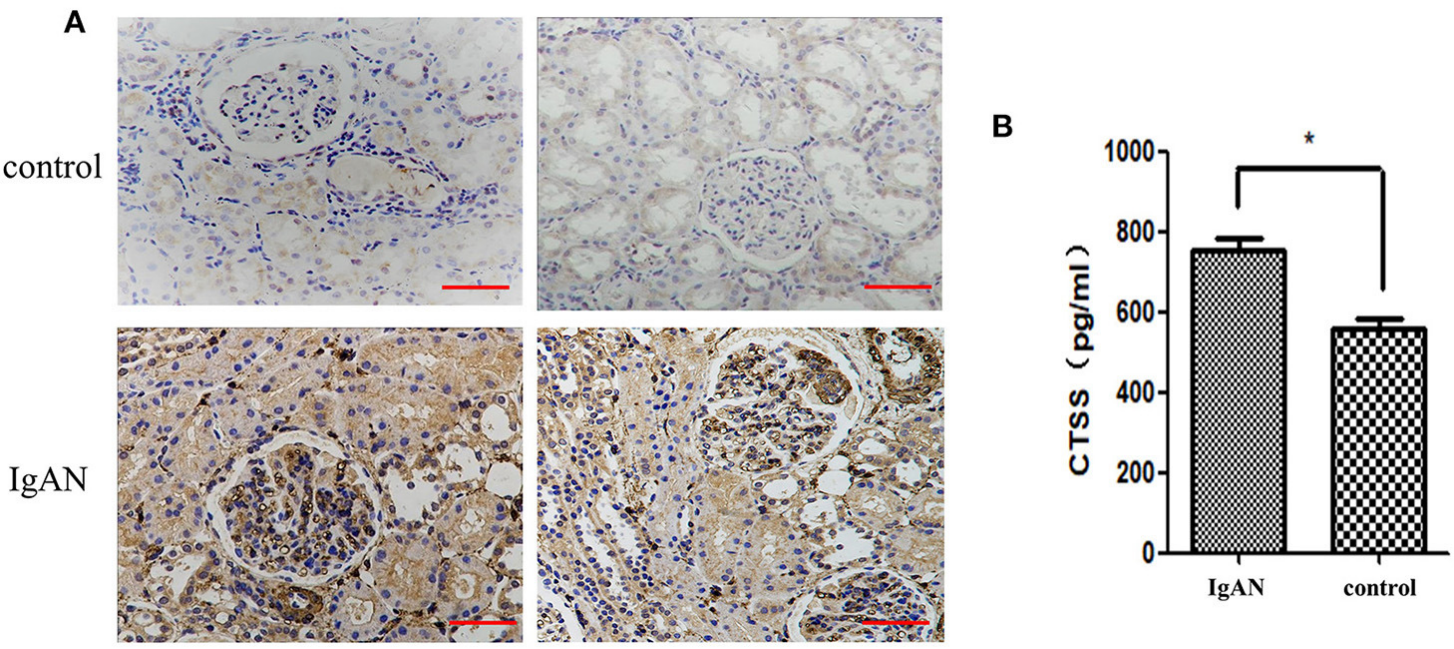
Higher levels CTSS are detected in renal tissues and serum samples of children with IgAN. **(A)** Immunohistochemical analysis of CTSS in human renal tissues. Scale bar = 50 μm. *N* = 25 patients, *N* = 8 control non-IgAN patients. **(B)** ELISA analysis of serum CTSS (*N* = 25 for IgAN patients and healthy children, respectively). Data are representative images (magnification ×400) or expressed as means ± SD of each group from at least three separate experiments. *P < 0.05 vs. the controls.

To determine the potential role of CTSS in the pathogenesis of IgAN, we recruited 25 children with IgAN and 25 age and gender-matched healthy children as well as eight patients with trauma or tumor (Table 1; Supplementary Tables 1, 2). Immunohistochemistry analysis indicated that anti-CTSS staining in the glomerular mesangium and tubular epithelial cells of biopsied renal tissues from 25 IgAN patients were remarkably stronger than that in those from non-IgAN patients with trauma or tumor (Figure 1A). ELISA revealed that the levels of serum CTSS in 25 IgAN patients, including 19 with Lee's classification (I + II), 6 with Lee's classification (III + IV), were significantly higher than that in healthy subjects in this population, and the serum CTSS levels in (III + IV) IgAN patients were significantly higher than that in (I + II) IgAN patients (*P* < 0.05, Figure 1B). Hence, increased levels of CTSS expression were detected in renal tissues and serum samples from IgAN patients.

### Serum CTSS Levels Are Positively Correlated With the Levels of 24-H-Urine Proteins, Urinary Microalbumin, Urine Erythrocytes, and the Grades of IgAN Lee's Classification in Children With IgAN

**Figure 2:**
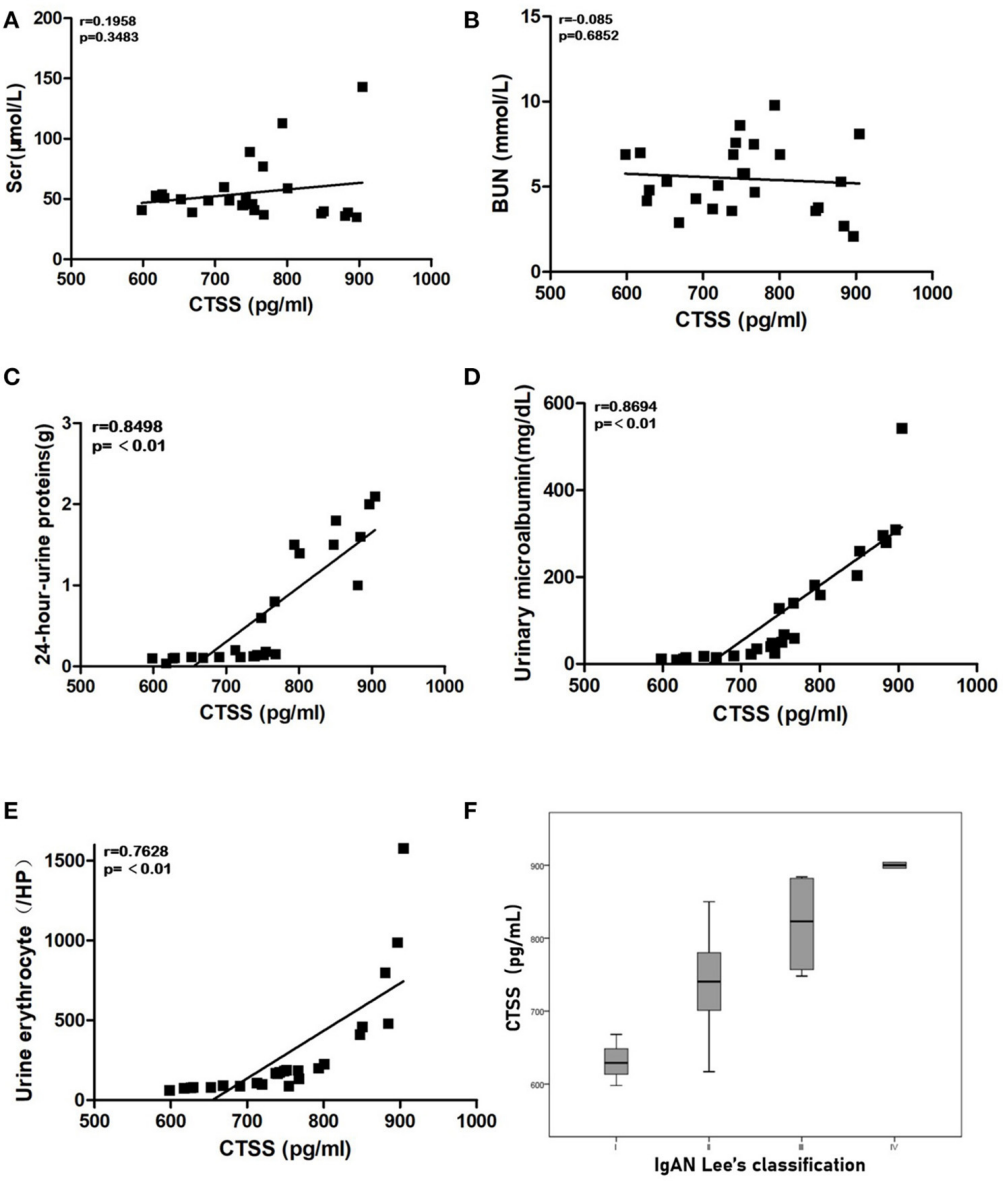
The levels of serum CTSS are correlated with clinical measures in IgAN patients. The potential correlation between the levels of serum CTSS and the values of each clinical measure were analyzed by Pearson-correlation coefficient (*r*). Data are the mean values from individual patients. **(A)** The levels of serum Scr. **(B)** The levels of serum BUN. **(C)** The levels of 24-h-urine proteins. **(D)** The levels of urinary microalbumin. **(E)** The numbers of urine erythrocytes in each high power field. **(F)** Stratification analysis of CTSS levels in different groups of IgAN patients.

Next, we analyzed the potential association between serum CTSS levels and some clinical measures in 25 IgAN patients. We found the serum CTSS levels were not associated with serum Scr and BUN levels in IgAN patients (Figures 2A,B). However, the levels of serum CTSS were positively correlated with the levels of 24-h-urine proteins (*R* = 0.79, *P* < 0.01), urinary microalbumin (*R* = 0.5822, *P* < 0.01) and urine erythrocytes (*R* = 0.7628, *P* < 0.01) in IgAN patients (Figures 2C–E). The levels of serum CTSS were positively associated with the grades of IgAN Lee's classification (*R* = 0.77, *P* < 0.01; Figure 2F). The significant correlation between serum levels of CTSS and these clinical measures suggest that up-regulated CTSS expression may contribute to the pathogenesis of IgAN.

### UP-Regulated CTSS Expression Occurs in Renal Tissues of IgAN Rats

**Figure 3:**
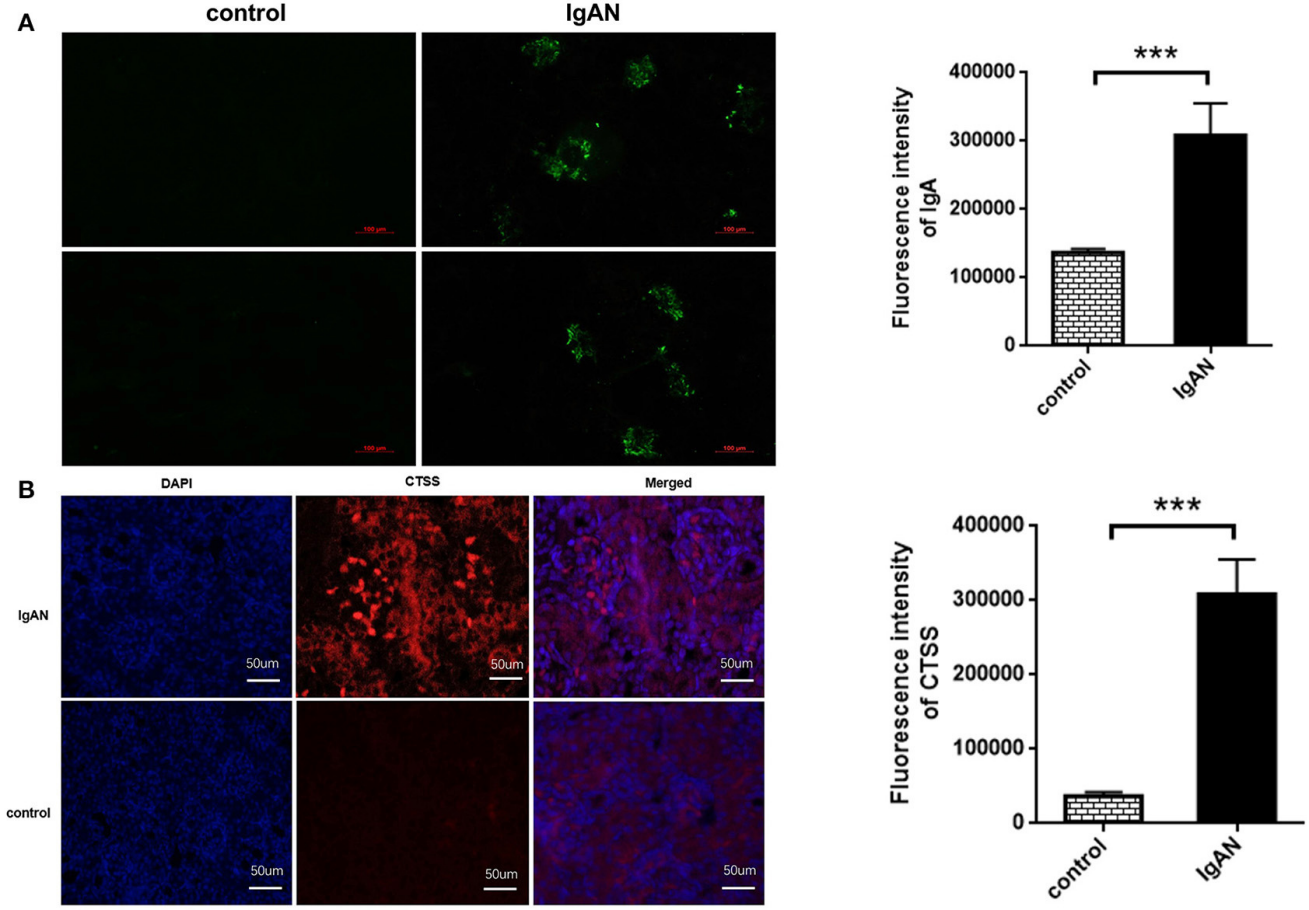
Immunofluorescent analysis of IgA accumulation and CTSS expression in the kidney of rats. Following induction of IgAN in rats, the levels of IgA accumulation and CTSS expression in the kidney tissues of experimental and control rats were determined by immunofluorescence. Data are representative images or present as the means ± SD of each group (*n* = 10 per group) from three separate experiments. **(A)** IgA accumulation in the mesangium of IgAN rats. **(B)** Up-regulated CTSS expression in the kidney of IgAN rats. ***P < 0.001 vs. the control.

To explore the role of CTSS in the development of IgAN, SD rats were induced for development of IgAN. Immunofluorescence displayed that IgA deposited in the glomeruli of IgAN rats, but little in the renal tissues of control rats, and quantitative analysis indicated that the areas of anti-IgA staining in the IgAN rats were significantly larger than that in the control rats (*P* < 0.01; Figure 3A). Further immunofluorescence revealed that the levels of CTSS expression in the glomeruli of IgAN rats were significantly higher than that in the controls (*P* < 0.01; Figure 3B). Thus, induction of IgAN significantly promoted the accumulation of IgA and increased the levels of CTSS expression in renal tissues of rats.

### Inhibition of CTSS Reduces Glomerular Mesangial Cell Proliferation *in vivo*

**Figure 4:**
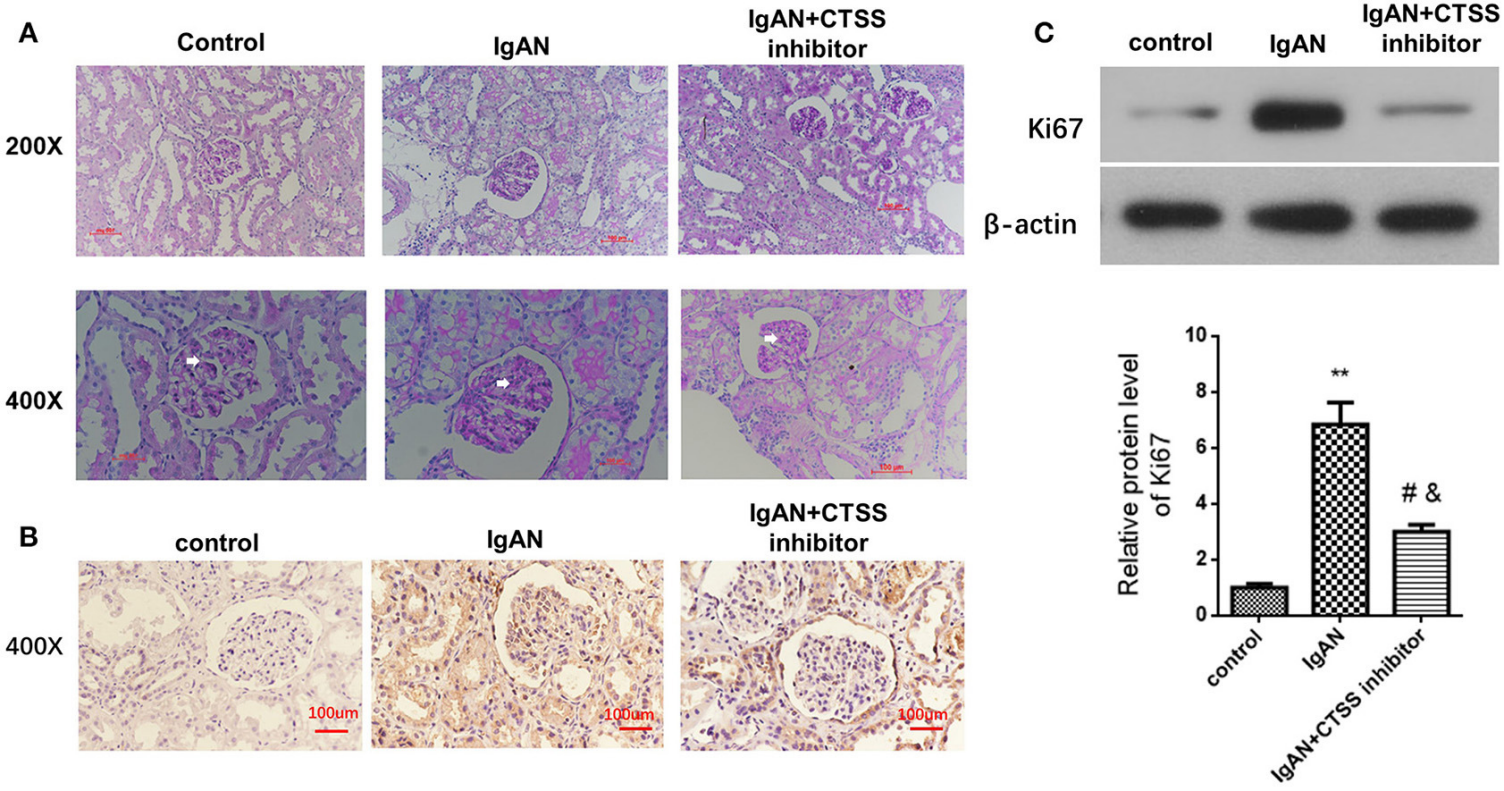
Treatment with a CTSS inhibitor reduces mesangial cell proliferation in IgAN rats. Following induction of IgAN and treatment with the CTSS inhibitor in rats, the pathological changes in the kidney tissues from the experimental and control groups of rats were analyzed PAS staining. The relative levels of Ki67 expression in renal tissues of each group of rats were determined by immunohistochemistry and Western blotting assays. Data are representative images or expressed as the means ± SD of each group (*n* = 10 per group) from three separate experiments. **(A)** PAS staining analysis of the kidney sections. **(B)** Immunohistochemistry analysis of Ki67 protein expression in kidney tissues. **(C)** Western blot analysis of the relative levels of Ki67 expression in the renal tissues **P < 0.01 vs. the Controls; ^#^*P* < 0.05 vs. the IgAN group; ^&^*P* < 0.05 vs. the Controls.

Glomerular mesangial cell proliferation is a pathogenic hallmark of IgAN. Next, we measured whether inhibition of CTSS could modulate the levels of Ki76 expression in renal tissues of different groups of rats. As shown in Figure 4A, PAS staining exhibited that there was no abnormality in the glomerular basilar membrane and mesangium, or no glomerular sclerosis in the control rats. In contrast, there was a slight increase in glomerular surface area, basement membrane thickness, accompanied by the mesangial expansion and some degrees of inflammatory infiltrates in the IgAN group. After treatment with CTSS inhibitor, there was no clear proliferative glomerular mesangium and basilar membrane and only minimal inflammatory infiltrates in the kidney tissues. Immunohistochemistry revealed that the levels of anti-Ki67 staining in the rats with a CTSS inhibitor were obviously less than that in the IgAN rats, but remained higher than that in the controls (Figure 4B). Western blot analysis displayed that the relative levels of Ki67 expression in the kidneys from the IgAN + CTSS inhibitor group were significantly lower than that in the IgAN group (*P* < 0.01) but still higher than that in the controls (*P* < 0.05; Figure 4C). Such three lines of data suggest that treatment with a CTSS inhibitor may inhibit glomerular mesangial cell proliferation in IgAN rats.

### Treatment With a CTSS Inhibitor Reduces the Proliferation of HMCs *in vitro*

**Figure 5:**
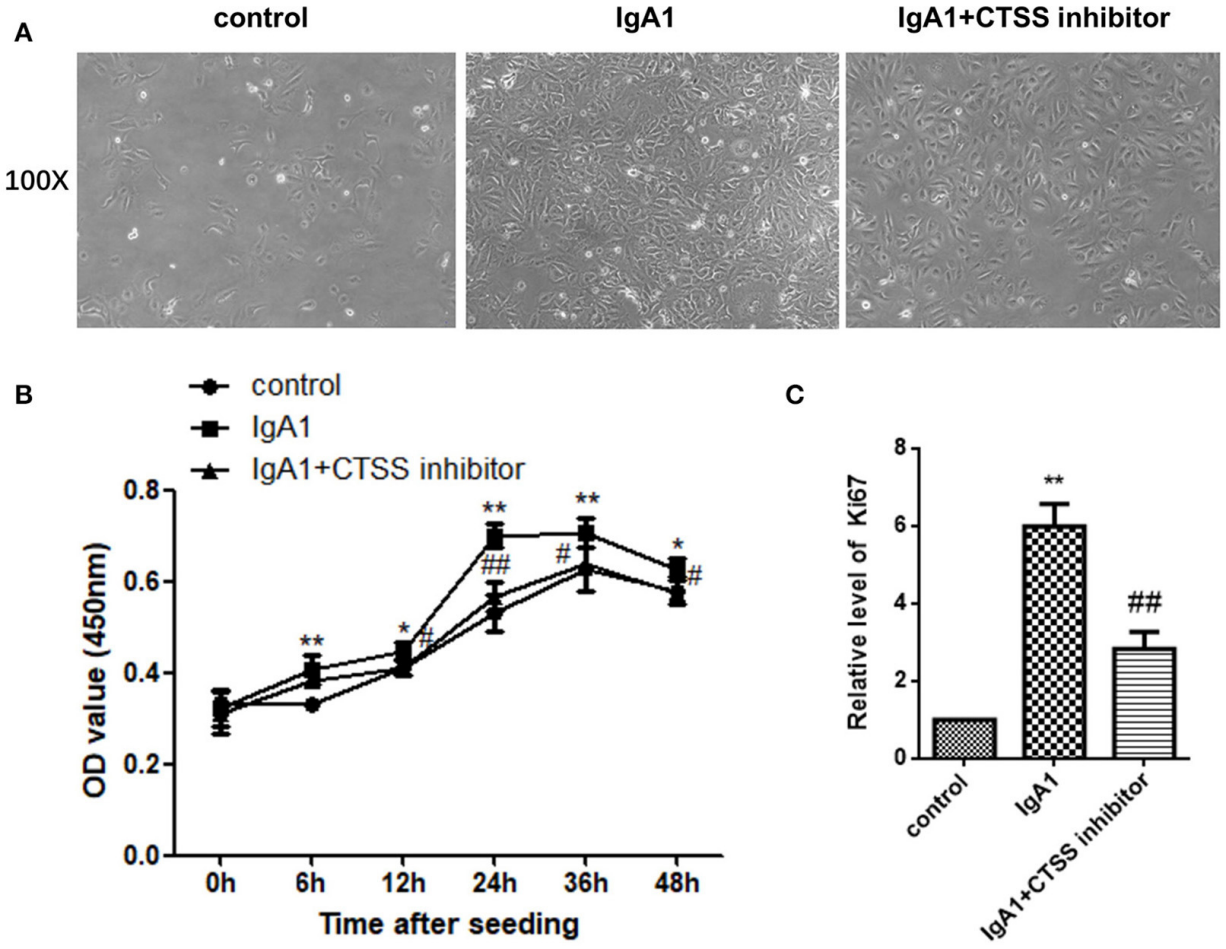
Treatment with the CTSS inhibitor mitigates the IgA1-stimulated the proliferation of HMCs *in vitro*. HMCs were pre-treated with vehicle or the CTSS inhibitor overnight and stimulated with, or without, IgA1 for the indicated time periods. Their morphology was observed under a light microscope and their cell viability was determined by CCK-8 assay. The relative levels of Ki67 to GAPDH mRNA transcripts were examined by qRT-PCR. Data are representative images or expressed as the means ± SD of each group from three separate experiments. **(A)** Morphological changes of cells. **(B)** CCK-8 analysis of cell viability. **(C)** Quantitative RT-PCR analysis of Ki67 mRNA transcripts in the cells. *P < 0.05, **P < 0.01 vs. the Controls; ^#^*P* < 0.05, ^##^*P* < 0.01 vs. the cells without the CTSS inhibitor.

Finally, we tested the impact of treatment with a CTSS inhibitor on the proliferation of HMCs *in vitro*. HMCs were pre-treated with, or without, the CTSS inhibitor overnight and treated in triplicate with, or without, IgA1 aggregates for varying time periods. The proliferation of HMCs was determined by CCK-8 assays. In comparison with the control cells without any treatment, treatment with IgA1 alone increased HMC clusters, while treatment with the CTSS inhibitor appeared slightly reduced the clusters of HMCs (Figure 5A). Longitudinal analyses indicated the numbers of HMCs following treatment with the CTSS inhibitor were only slightly greater than that in the controls, but significantly less than that in the IgA1-treated alone group, particularly after treatment with the CTSS inhibitor for 24–48 h (Figure 5B). Further analyses revealed that the relative levels of Ki67 mRNA transcripts to the control GAPDH in the CTSS inhibitor-treated cells were significantly lower than that in the cells treated with IgA1 alone, but remained higher than that of the controls (Figure 5C). Such data demonstrated that treatment with the CTSS inhibitor significantly inhibited the IgA1-stimulated HMC proliferation *in vitro*.

## Discussion

CTSS is a stable cysteine protease with both intra and extracellular activity (14–16). CTSS acts as a processing enzyme to regulate protein trafficking and secretion, and extracellularly modulates tissue remodeling (15, 17–19). As an important cysteine protease, CTSS participates in the pathogenesis of autoimmune diseases, allergic inflammation and asthma, diabetes, obesity, cardiovascular and pulmonary diseases as well as cancer by promoting tumor invasiveness or acting as a biomarker for evaluating cardiovascular diseases (20–22). Furthermore, inflammatory IL-1β, and IFN-γ can stimulate vascular smooth muscle cells to secrete CTSS that degrades insoluble elastin and disrupts homeostasis of atherosclerotic plaque (22). Inhibition of CTSS activity can decrease incidence of smoking-related chronic obstructive pulmonary disease (COPD) (23). In addition, treatment with a CTSS inhibitor decreases the severity of lupus nephritis in animals by inhibiting CTSS activity and antigen presenting cell activity to attenuate the MHC II-related lymphocyte responses (24) and CTSS knockout decreases the risk for development of diabetic nephropathy by reducing microvascular complications (25, 26). Moreover, increased levels of plasma CTSS are negatively correlated with glomerular filtration rates in CKD mice and positively with disease progression (27, 28). Similarly, the levels of serum CTSS are positively correlated with the degrees of angiogenesis and inflammation in patients with ESRD (29) while treatment with the CTSS inhibitor can reduce cardiovascular inflammation in patients with CKD (30). It is reported that CTSS can promote cell proliferation. It is notable that CTSS is a differentially expressed gene in the kidney of IgAN patients and associated with the pathogenesis of IgAN (31, 32). In this study, we found significantly up-regulated CTSS expression in the kidney tissues, particularly in the glomerular mesangium and tubular epithelial cells from human IgAN patients, and higher levels of serum CTSS were positively correlated with the levels of 24-h-urine proteins and microalbumin and urine erythrocytes in IgAN patients in this population. Such data extended previous observations and support the notion that up-regulated CTSS participates in the pathogenesis of IgAN (31, 32). Given that the concentrations of 24-h-urine proteins and microalbumin as well as urine erythrocytes are valuable measures for pathogenesis of IgAN. Interestingly, there appeared to be a cutoff at CTSS 750 pg/ml, above which the levels of 24-h-urine proteins and microalbumin and urine erythrocytes increased in this population. This population included the majority (76%) of patients with IgAN at mild grades (I + II) of Lee's classification and those patients displayed CTSS levels of <750 pg/ml while patients with high grades (III or IV) of Lee's classification exhibited higher levels of CTSS (>750 pg/ml). If there were more patients with high-grades of IgAN the correlation between the levels of serum CTSS and the grades of Lee's classification should be more significant. The significant correlations of these measures with serum CTSS levels and between serum CTSS levels and IgAN pathological grades in this population suggest that serum CTSS levels may be useful for evaluating the severity of IgAN. However, the diagnostic value of serum CTSS levels needs to be further validated in a bigger population with more patients with high grades of IgAN.

How does CTSS contribute to the pathogenesis of IgAN? A number of clinical and laboratory studies have shed lights on the function of CTSS in promoting the proliferation of tumor cells. Hence, inhibition of CTSS may inhibit the proliferation of vascular endothelial and glomerular mesangial cells, ameliorating the pathogenic process of IgAN. We employed a rat model of IgAN using an available protocol (33). We found that induction of IgAN caused IgA glomerular accumulation and enhanced CTSS expression in the kidney of rats, accompanied by increased glomerular surface area, basement membrane thickness, the mesangial expansion, and some degrees of inflammatory infiltrates. As the Ki67 protein is widely used as a proliferation marker (34) we explored how up-regulated CTSS expression was associated with cell proliferation in the glomeruli. Interestingly, high levels of Ki67 expression were detected in the kidney of IgAN rats, suggesting that increased CTSS was associated with cell proliferation, like the glomerular mesangial cell proliferation, a hallmark of IgAN pathogenesis. Furthermore, treatment with LY3000328 significantly mitigated the IgAN-related pathogenic alternations in the glomerular mesangium and significantly reduced the levels of Ki67 expression in the kidney of rats. More importantly, treatment with LY3000328 also significantly decreased the IgA1-stimulated proliferation of HMCs, evidenced by decreased cell viability and Ki67 expression, consistent with previous observations (35, 36). It was notable that treatment with LY3000328 did not abrogate the IgA1-stimulated proliferation of HMCs. This suggest that CTSS may support the proliferation of HMCs. However, CTSS is not an only factor to regulate the proliferation of HMCs and some other factors may also promote the proliferation of HMCs. We are interested in further investigating which and how other factors regulate the proliferation of HMCs and how they interact with CTSS.

Mesangial cell proliferation is a key histological marker in many renal diseases, including IgAN, mesangial proliferative lupus glomerulonephritis, membranous proliferative glomerulonephritis, and diabetic nephropathy (37). It is possible that IgA1 binds to the receptors, such as transferrin receptor TfR and FcRα/μ, on glomerular mesangial cells to activate the nuclear transcription factor (NF-κB) and ERK signaling (16, 18). As a result, these promote the secretion of growth factor and cytokines, such as interleukin (IL)-6, IL-8, IL-1, tumor necrosis factor (TNF)-α, monocyte chemoattractant protein (MCP)-1, platelet activating factor (PAF), macrophage migration inhibitory factor (MIF), which enhance mesangial cell proliferation in an autocrine or paracrine manner (38–40). Excessive proliferation of mesangial cells would produce high levels of cytokines and extracellular matrix, positively-feedback to mediate glomerular sclerosis and interstitial fibrosis. Apparently, CTSS may be crucial for the IgA1-mediated glomerular mesangial cell proliferation and CTSS may be a potential therapeutic target for design of new strategies for intervention of IgAN.

We recognized that our study had limitations, such as a small sample size and most cases with mild grades [Lee's classification (I + II)] of IgAN, which led to low levels of crescents and low percentages of sclerosis, and the lack of measurements of inflammatory cytokines and chemokines. The less diversity of population may also lead to no statistical significance between the levels of serum CTSS and crescents or percentages of crescents in this population. Therefore, future investigations with a bigger population with diversity of IgAN patients are warranted to validate the findings from this study and to determine the precise role of CTSS in the development and progression of IgAN.

In summary, our data indicated significantly up-regulated CTSS expression in the kidney of IgAN patients and that higher levels of serum CTSS were positively correlated with the levels of 24-h-urine proteins and microalbumin as well as urine erythrocytes in IgAN patients. Similarly, induction of IgAN induced higher levels of IgA accumulation in the glomeruli, CTSS and Ki67 expression in the kidney of rats, accompanied by obviously renal pathological changes. Treatment with LY3000328 significantly mitigated the IgAN-related pathogenic alternations in the glomerular mesangium and significantly reduced the levels of Ki67 expression in the kidney of rats. Moreover, treatment with LY3000328 significantly decreased the IgA1-stimulated glomerular mesangial cell proliferation and Ki67 expression in HMCs *in vitro*. Hence, CTSS may be crucial for the IgA-mediated glomerular mesangial cell proliferation, contributing to the pathogenesis of IgAN. Therefore, serum CTSS level may be valuable for evaluating the severity of IgAN and a potential therapeutic target for intervention of IgAN.

## Data Availability Statement

The raw data supporting the conclusions of this article will be made available by the authors, without undue reservation.

## Ethics Statement

The studies involving human participants were reviewed and approved by the Ethics Committee of Shengjing Hospital, China Medical University (2019PS441K). Written informed consent to participate in this study was provided by the participants' legal guardian/next of kin. The animal study was reviewed and approved by the Ethics Committee of Shengjing Hospital, China Medical University (2019PS441K).

## Author Contributions

JZ and YY: conception and design of the study and collection of data and writing the manuscript. YW: conception and design of the study and analysis of data. All authors contributed to the article and approved the submitted version.

## Conflict of Interest

The authors declare that the research was conducted in the absence of any commercial or financial relationships that could be construed as a potential conflict of interest.
