# Supplementary material for: The Clinical Significance and Potential Role of Cathepsin S in IgA Nephropathy
Source: Front Pediatr. 2021 Apr 12;9:631473. doi: 10.3389/fped.2021.631473 (PMC8071879; doi:10.3389/fped.2021.631473)
Supplement: Supplementary file 1 [file Table_1.DOCX]

| Gender | Age  (yrs) | CTSS (pg/ml) | Scr  (μmol/L) | BUN  (mmol/L) | 24-hour-urine proteins(g) | Urinary microalbumin (mg/dL) | Urine erythrocytes/HP | IgAN Lee’s classification | Numbers of crescents | % of crescents |
| --- | --- | --- | --- | --- | --- | --- | --- | --- | --- | --- |
| M | 9 | 751 | 46 | 5.8 | 0.14 | 50 | 188 | II | 0 | 0 |
| M | 7 | 737 | 45 | 3.6 | 0.13 | 40 | 170 | II | 1 | 6.7 |
| F | 12 | 629 | 51 | 4.8 | 0.11 | 15 | 81 | I | 0 | 0 |
| M | 10 | 719 | 49 | 5.1 | 0.12 | 35 | 99 | II | 2 | 4.7 |
| F | 9 | 800 | 59 | 6.9 | 1.4 | 159 | 227 | II | 2 | 5 |
| M | 11 | 850 | 40 | 3.8 | 1.8 | 260 | 460 | II | 0 | 0 |
| F | 12 | 668 | 39 | 2.9 | 0.11 | 15 | 91 | I | 0 | 0 |
| M | 12 | 896 | 35 | 2.1 | 2 | 309 | 989 | IV | 7 | 15 |
| F | 11 | 767 | 37 | 4.7 | 0.15 | 59 | 134 | II | 3 | 5 |
| F | 9 | 739 | 45 | 6.9 | 0.13 | 48 | 167 | II | 3 | 8 |
| M | 8 | 766 | 97 | 7.5 | 0.8 | 140 | 187 | III | 4 | 12 |
| M | 6 | 748 | 98 | 8.6 | 0.6 | 128 | 183 | III | 5 | 20 |
| F | 14 | 880 | 36 | 5.3 | 1 | 296 | 798 | III | 3 | 8 |
| F | 13 | 617 | 53 | 7 | 0.04 | 10 | 77 | II | 3 | 13 |
| M | 12 | 626 | 54 | 4.2 | 0.1 | 12 | 78 | II | 4 | 16 |
| F | 14 | 884 | 39 | 2.7 | 1.6 | 280 | 481 | III | 4 | 8 |
| M | 11 | 754 | 41 | 5.8 | 0.18 | 68 | 90 | II | 1 | 5 |
| M | 9 | 742 | 50 | 7.6 | 0.14 | 25 | 175 | II | 2 | 3.8 |
| F | 12 | 904 | 143 | 8.1 | 2.1 | 543 | 1578 | IV | 8 | 16 |
| M | 11 | 598 | 91 | 6.9 | 0.1 | 12 | 62 | I | 0 | 0 |
| F | 8 | 652 | 50 | 5.3 | 0.12 | 18 | 80 | II | 4 | 19 |
| M | 9 | 847 | 38 | 3.6 | 1.5 | 204 | 412 | II | 0 | 0 |
| M | 12 | 712 | 80 | 3.7 | 0.2 | 23 | 109 | II | 0 | 0 |
| M | 11 | 793 | 113 | 9.8 | 1.5 | 182 | 201 | II | 1 | 6 |
| M | 13 | 690 | 49 | 4.3 | 0.12 | 19 | 90 | II | 3 | 14 |

Supplementary Table 1. The demographic and clinical data of 25 children with IgAN
