# Supplementary material for: The Clinical Significance and Potential Role of Cathepsin S in IgA Nephropathy
Source: Front Pediatr. 2021 Apr 12;9:631473. doi: 10.3389/fped.2021.631473 (PMC8071879; doi:10.3389/fped.2021.631473)
Supplement: Supplementary file 2 [file Table_2.DOCX]

Supplementary Table 2. The demographic and clinical data of eight non-IgA patients.

| Gender | Age(yrs) | Scr(μmol/L) | BUN (mmol/L) |
| --- | --- | --- | --- |
| M | 9 | 49 | 3.8 |
| F | 10 | 46 | 5.6 |
| M | 9 | 50 | 2.8 |
| F | 11 | 50 | 5.1 |
| M | 12 | 54 | 6 |
| M | 10 | 43 | 3.8 |
| F | 11 | 52 | 5.7 |
| M | 10 | 47 | 3.1 |
